# Comparative analysis of vancomycin-resistant enterococci in colonization and infection—a longitudinal study

**DOI:** 10.1128/spectrum.01750-25

**Published:** 2025-10-16

**Authors:** Vera Blaschke, Vera Rauschenberger, Heike Claus, Stefanie Kampmeier

**Affiliations:** 1 University of Würzburg, Institute for Hygiene and Microbiology 9190, Würzburg, Germany; 2 University Hospital Würzburg, Infection Control and Antimicrobial Stewardship Unit 27207, Würzburg, Germany; Istituto Dermatologico San Gallicano, Rome, Italy

**Keywords:** vancomycin resistance, VRE, intestinal colonization, bloodstream infections

## Abstract

Vancomycin-resistant *Enterococcus faecium* (VREfm) carriage in the gastrointestinal tract is a risk factor for the development of an invasive infection. The exact mechanisms underlying the transition from colonization to infection are still unclear. We conducted a longitudinal study, including 54 paired VREfm isolates, consisting of a colonization and a subsequent bloodstream isolate from the same patient. We performed whole-genome sequencing, biofilm formation assays, and spot-on-lawn assays to investigate genotypic and phenotypic characteristics of the isolates. No significant differences in these characteristics between paired colonization and infection isolates were detected. Genotyping revealed that colonization isolates were genetically closely related to their respective infection isolates in 22 of 27 (81%) isolate pairs. Further studies focusing on the interaction between host epithelium and pathogen are needed to gain more insight into the transition from colonization to infection.

**IMPORTANC**E**:**

Previous studies have primarily focused on patient-related risk factors associated with the development of vancomycin-resistant *Enterococcus faecium* (VREfm) infection. However, identifying and characterizing the bacterial factors responsible for this transition is crucial, especially given the limited treatment options for VREfm infection. Our analyses revealed no significant differences between colonization and infection isolates, suggesting that host-pathogen interaction may play a more critical role in this progression and should be further investigated. Moreover, our findings highlight the importance of risk assessment and infection prevention measures to prevent VREfm colonization as a critical step in the development of VREfm infection.

## INTRODUCTION

Enterococci are part of the normal human gut microbiota. However, these pathogens can cause severe infections, such as bloodstream infections (BSI), especially in critically ill or immunocompromised patients. In particular, *Enterococcus faecium* with acquired vancomycin resistance (vancomycin-resistant *E. faecium* [VREfm]) hampers successful and targeted antibiotic treatment in case of an infection (1). In most patients, VREfm infection is preceded by colonization (2). Risk factors for colonization comprise antibiotic therapy, frequent contact with health care, long-term hospitalization, immunosuppression, and admission to intensive care units (3). Risk factors for VREfm infection comprise previous colonization with VREfm, allogeneic bone marrow transplantation, neutropenia, or central venous catheterization (4). While these host-associated risk factors are well studied, pathogen-associated factors that may trigger VREfm infection in colonized hosts are largely unknown. Chilambi et al. investigated genomic and phenotypic changes in VREfm during intestinal colonization and BSI of immunocompromised pediatric patients in a retrospective cohort study and identified mutations affecting bacterial growth and biofilm formation as crucial for infection development (5). Recently, bacteriocins were identified to play an important role in inter- and intraspecies interactions in enterococci (6–8). Up to now, data addressing genotypic and phenotypic differences of VREfm originating from colonization and infection in adult patients are scarce. The present retrospective cohort study aims to identify differences in colonization and infection isolates and their interaction to understand the transition leading to human infection.

## RESULTS

### Patient cohort

The present study comprised a cohort of 27 patients from a tertiary hospital in Germany who had initially been colonized with VREfm and subsequently developed a VREfm BSI (Table 1). The cohort consisted of 20 male (74%) and 7 female (26%) patients, with an age range of 23 to 81 years (median age = 60 years). The interval between rectal colonization and the onset of BSI with VREfm ranged between 5 and 1,836 days (average = 313 days). The majority of patients were diagnosed with an oncological disease (*n* = 20; 74%), with hematological-oncological disease being the most prevalent (*n* = 16; 59%). The remaining patients (*n* = 7; 26%) were diagnosed with other underlying diseases (including hepatitis C [*n* = 2; 7%], pancreatitis [*n* = 2; 7%], and peripheral arterial disease [*n* = 1; 4%]). Four patients (18%) died within 14 days after detection of VREfm in the blood culture, which we defined as a BSI-associated death. No significant differences in patient outcome and presence of virulence factors could be detected regarding biofilm formation or bacteriocin production. Further information regarding the correlation of detected virulence factors and BSI-associated mortality is provided in Table 2.

**TABLE 1:** Patient information: age, sex, and underlying disease^*a*^

Patient	Age (years)	Sex	Underlying disease	Time between colonization and infection (days)
1	55	M	Hepatitis C	498
2	67	M	Acute myeloid leukemia	1,836
3	60	M	Pancreatic cancer	8
4	76	F	Neuroendocrine tumor with liver metastases	294
5	81	M	No primary disease	8
6	67	M	Diffuse large B cell lymphoma	110
7	56	M	Angioimmunoblastic T cell lymphoma	16
8	70	M	Acute myeloid leukemia	9
9	48	M	B cell lymphoma	1,638
10	24	F	Diffuse large B cell lymphoma	27
11	59	F	Angioimmunologic T-NHL	307
12	23	M	Biphenotypic acute leukemia	23
13	73	F	Anal cancer	7
14	69	M	Diffuse large B cell lymphoma	5
15	36	M	Chronic pancreatitis	1,146
16	70	M	Post-kidney transplantation	78
17	68	M	Peripheral artery disease	9
18	67	M	Acute myeloid leukemia	59
19	42	F	Pancreatic neuroendocrine tumor	11
20	53	M	Nodular lymphocyte-predominant Hodgkin lymphoma	60
21	19	M	B cell acute lymphoblastic leukemia	527
22	74	M	Rheumatoid arthritis (MTX therapy)	12
23	51	M	Angioimmunoblastic T-NHL	560
24	48	F	Acute myeloid leukemia	320
25	60	M	B cell acute lymphoblastic leukemia	195
26	52	F	Acute myeloid leukemia	245
27	63	M	Hepatitis C	439

^*a*^ Time between colonization and infection.

**TABLE 2:** Analyzed virulence factors of colonization (a) and infection (b) isolates and clinical outcome of patients

Patient	Isolate	Biofilm formation	Bacteriocin production	BSI-associated mortality^*a*^
1	1a	Yes	bac43, entA	Yes
	1b	No	bac43, entA	
2	2a	No	bac43, entA	No
	2b	No	bac43, entA	
3	3a	No	bac43, entA	No
	3b	No	bac43, entA	
4	4a	No	bac43, entA, bac51	No
	4b	No	bac43, entA, bac51	
5	5a	Yes	bac43, entA	Yes
	5b	No	bac43, entA	
6	6a	No	bac43, entA	No
	6b	No	bac43, entA	
7	7a	No	bac43, entA, bac51	No
	7b	No	bac43, entA, bac51	
8	8a	No	bac43, entA, bac51	No
	8b	No	bac43, entA, bac51	
9	9a	No	bac43	No
	9b	No	bac43, entA	
10	10a	No	bac43, entA	No
	10b	No	bac43, entA	
11	11a	No	bac43, entA	No
	11b	No	bac43, entA, bac51	
12	12a	No	bac43, entA	No
	12b	Yes	bac43, entA	
13	13a	No	bac43, entA	Yes
	13b	No	bac43, entA	
14	14a	No	bac43, entA, bac51	No
	14b	No	bac43, entA, bac51	
15	15a	Yes	bac43, entA	No
	15b	No	bac43, entA, bac51	
16	16a	No	bac43, entA, bac51	No
	16b	No	bac43, entA, bac51	
17	17a	No	bac43, entA	No
	17b	No	bac43, entA	
18	18a	No	entA	No
	18b	No	entA	
19	19a	No	bac43, entA, bac51	No
	19b	No	bac43, entA, bac51	
20	20a	No	bac43, entA	No
	20b	No	bac43, entA	
21	21a	No	bac43, entA, bac51	No
	21b	No	bac43, entA, bac51, bac32	
22	22a	No	bac43, entA, bac51	No
	22b	No	bac43, entA, bac51	
23	23a	No	bac43, entA	Yes
	23b	No	bac43, entA, bac51	
24	24a	No	bac43, entA	No
	24b	No	bac43, entA	
25	25a	No	bac43, entA	No
	25b	Yes	bac43, entA	
26	26a	No	bac43, entA	No
	26b	No	entA, bac51	
27	27a	No	bac43, entA	No
	27b	No	bac43, entA, bac51	

^*a*^ Defined as 14 days mortality after positive blood culture sample and clinical signs of infection.

### Molecular characteristics of VREfm isolate

**Fig 1:**
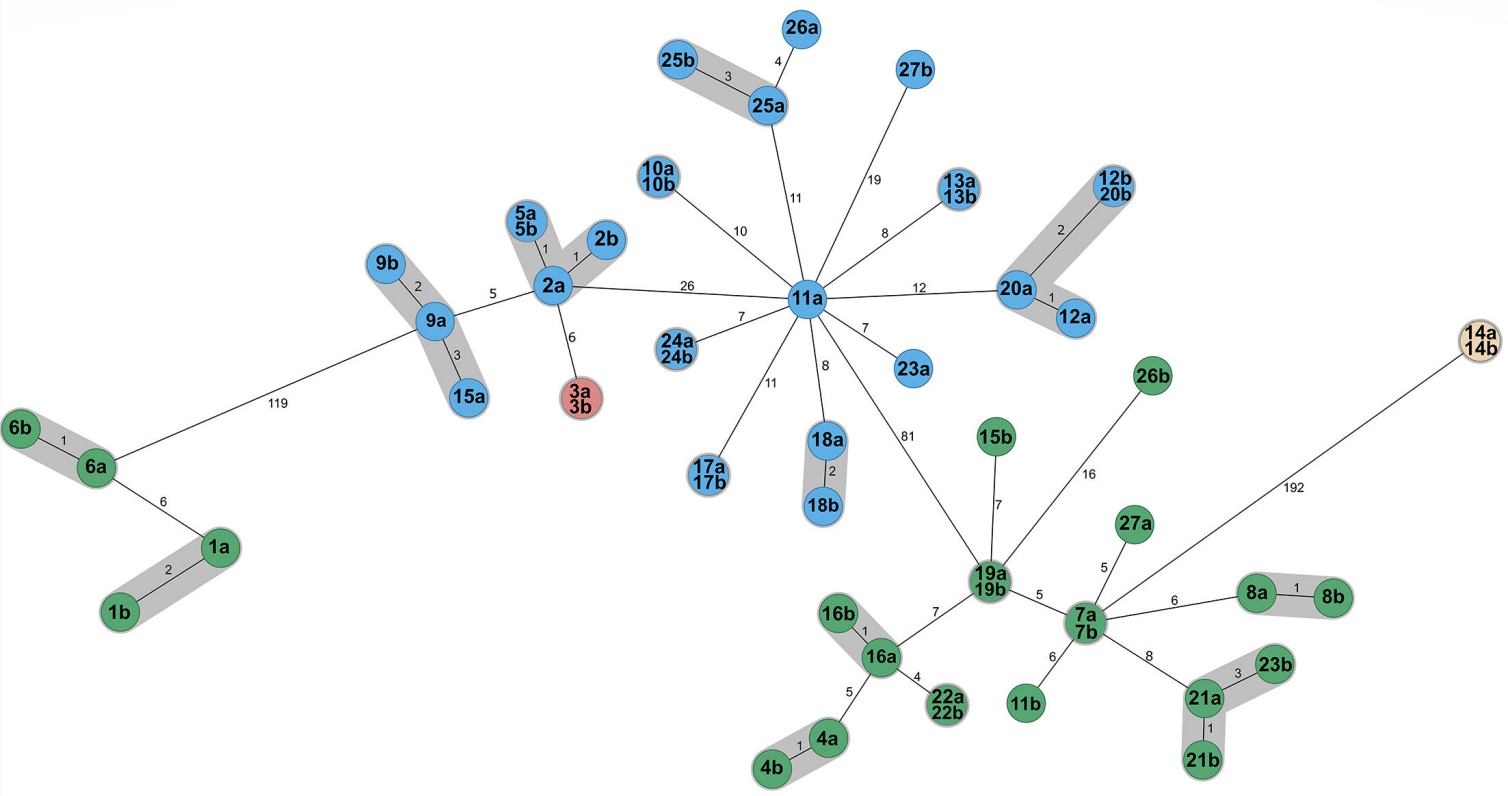
Minimum spanning tree of VREfm colonization (labeled a) and infection (labeled b) isolates displaying results of the cgMLST analysis. *In silico* extracted MLST STs are represented by different colors: ST117 (blue), ST80 (green), ST2032 (red), and ST2542 (beige). The number of differing alleles is indicated in black on the connecting lines, which serve to illustrate the genetic similarity between isolates. A close genetic relation is indicated in gray.

A total of 54 clinical isolates were investigated in this study, obtained between 2015 and 2023 from 27 patients. The genome sequencing demonstrated the presence of the *vanB* gene in all isolates. Multi-locus sequence typing identified four different sequence types (STs), i.e., ST117 (*n* = 27), ST80 (*n* = 23), ST2542 (*n* = 2), and ST2032 (*n* = 2). All STs belonged to clonal complex 17. In five patients, the ST of the colonizing isolate differed from that of the invasive isolate (pair 11, 15, 23, 26, 27). A summary of the molecular characteristics of the isolates is provided in Table 3. A minimum spanning tree based on the core genome multilocus sequence typing (cgMLST) indicates the allelic distances of the isolates (Fig. 1). Some isolates did not show any differences in the cgMLST analysis.

**TABLE 3:** Molecular characteristics, presence (1), and absence (0) of putative virulence factors in colonization and infection VREfm isolate pairs^*a*^

Patient	Strain	Origin	MLST ST	cgMLST CT	IS16-Entfm-hosp	acm-Entfm-hosp	bepA-Entfm-hosp	ccpA-Entls	ecbA-Entfm-hosp	empA-Entfm-hosp	empB-Entfm-hosp	empC-Entfm-hosp	fms11-Entfm-hosp	fms13-Entfm	fms14-Entfm-hosp	fms15-Entfm-hosp	fms16-Entfm-hosp	fms17-Entfm-hosp	fms19-Entfm-hosp	fms20-Entfm-hosp	fms21-Entfm-com	fnm-Entfm-hosp	gls20-Entfm	gls33-Entfm	glsB-Entfm	glsB1-Entfm	hylEntfm-Entfm-hosp	orf1481-Entfm-hosp	ptsD-Entfm-hosp	sagA-Entfm-hosp	scm-Entfm-com	sgrA-Entfm-hosp
1a	M5324	Rectal	80	1,724	1	1	1	1	1	1	1	1	1	1	1	1	1	1	1	0	0	1	1	1	1	1	1	1	1	1	1	1
1b	M7369	Blood	80	1,724	1	1	1	1	1	1	1	1	1	1	1	1	1	1	1	0	0	1	1	1	1	1	1	1	1	1	0	1
2a	M4923	Rectal	117	469	1	1	1	1	1	1	1	1	1	1	1	1	1	1	1	1	1	1	1	1	1	1	1	1	1	1	1	1
2b	M12038	Blood	117	469	1	1	1	1	1	1	1	1	1	1	1	1	1	1	1	1	1	1	1	1	1	1	1	1	1	1	0	1
3a	M11253	Rectal	2,032	469	1	1	1	1	1	1	1	1	1	1	1	0	1	1	1	0	0	1	1	1	1	1	1	1	1	1	1	1
3b	M11278	Blood	2,032	469	1	1	1	1	1	1	1	1	1	1	1	0	1	1	1	0	0	1	1	1	1	1	1	1	1	1	0	1
4a	M10885	Rectal	80	1,065	1	1	1	1	1	1	1	1	1	1	1	1	1	1	1	0	0	1	1	1	1	1	1	1	1	1	0	1
4b	M11870	Blood	80	1,065	1	1	1	1	1	1	1	1	1	1	1	1	1	1	1	0	0	1	1	1	1	1	1	1	1	1	0	1
5a	M9308	Rectal	117	469	1	1	1	1	1	1	1	1	1	1	1	1	1	1	1	1	1	1	1	1	1	1	1	1	1	1	1	1
5b	M9368	Blood	117	469	1	1	1	1	1	1	1	1	1	1	1	1	1	1	1	1	1	1	1	1	1	1	1	1	1	1	1	1
6a	M555R0	Rectal	80	1,724	1	0	1	1	1	1	1	1	1	1	1	1	1	1	1	0	0	1	1	1	1	1	1	1	1	1	0	1
6b	M5974	Blood	80	1,724	1	1	1	1	1	1	1	1	1	1	1	1	1	1	1	0	0	1	1	1	1	1	1	1	1	1	0	1
7a	M705R6	Rectal	80	1,065	1	1	1	1	1	1	1	1	1	1	1	1	1	1	1	0	0	1	1	1	1	1	1	1	1	1	1	1
7b	M7132	Blood	80	1,065	1	1	1	1	1	1	1	1	1	1	1	1	1	1	1	0	0	1	1	1	1	1	1	1	1	1	0	1
8a	M12355	Rectal	80	1,065	1	1	1	1	1	1	1	1	1	1	1	1	1	1	1	0	0	1	1	1	1	1	1	1	1	1	1	1
8b	M12375	Blood	80	1,065	1	1	1	1	1	1	1	1	1	1	1	1	1	1	1	0	0	1	1	1	1	1	1	1	1	1	1	1
9a	M3740	Rectal	117	469	1	1	1	1	1	1	1	1	1	1	1	0	1	1	1	0	0	1	1	1	1	1	1	1	1	1	1	1
9b	M10251	Blood	117	469	1	1	1	1	1	1	1	1	1	1	1	1	1	1	1	0	0	1	1	1	1	1	1	1	1	1	0	1
10a	M11988	Rectal	117	71	1	1	1	1	1	1	1	1	1	1	1	1	1	1	1	0	0	1	1	1	1	1	1	1	1	1	0	1
10b	M12072	Blood	117	71	1	1	1	1	1	1	1	1	1	1	1	1	1	1	1	0	0	1	1	1	1	1	1	1	1	1	1	1
11a	M6985	Rectal	117	71	1	1	1	1	1	1	1	1	1	1	1	1	1	1	1	0	0	1	1	1	1	1	1	1	1	1	0	1
11b	M8421	Blood	80	1,065	1	1	1	1	1	1	1	1	1	1	1	1	1	1	1	0	0	1	1	1	1	1	1	1	1	1	0	1
12a	M12188	Rectal	117	71	1	1	1	1	1	1	1	1	1	1	1	1	1	1	1	0	0	1	1	1	1	1	1	1	1	1	0	1
12b	M12247	Blood	117	71	1	1	1	1	1	1	1	1	1	1	1	1	1	1	1	0	0	1	1	1	1	1	1	1	1	1	1	1
13a	M9079	Rectal	117	71	1	1	1	1	1	1	1	1	1	1	1	1	1	1	1	0	0	1	1	1	1	1	1	1	1	1	1	1
13b	M9112	Blood	117	71	1	1	1	1	1	1	1	1	1	1	1	1	1	1	1	0	0	1	1	1	1	1	1	1	1	1	0	1
14a	M8362	Rectal	2,542	3,108	1	1	1	1	1	1	1	1	1	1	1	1	1	1	1	0	0	1	1	1	1	1	1	1	1	1	1	1
14b	M8392	Blood	2,542	3,108	1	1	1	1	1	1	1	1	1	1	1	1	1	1	1	0	0	1	1	1	1	1	1	1	1	1	1	1
15a	M3255	Rectal	117	469	1	0	1	1	1	1	1	1	1	1	1	1	1	1	1	0	0	1	1	1	1	1	1	1	1	1	0	1
15b	M7568	Blood	80	1,065	1	1	1	1	1	1	1	1	1	1	1	1	1	1	1	0	0	1	1	1	1	1	1	1	1	1	0	1
16a	M11222	Rectal	80	1,065	1	1	1	1	1	1	1	1	1	1	1	1	1	1	1	0	0	1	1	1	1	1	1	1	1	1	1	1
16b	M11514	Blood	80	1,065	1	1	1	1	1	1	1	1	1	1	1	1	1	1	1	0	0	1	1	1	1	1	1	1	1	1	0	1
17a	M8820	Rectal	117	71	1	1	1	1	1	1	1	1	1	1	1	1	1	1	1	0	0	1	1	1	1	1	1	1	1	1	0	1
17b	M8856	Blood	117	71	1	1	1	1	1	1	1	1	1	1	1	1	1	1	1	0	0	1	1	1	1	1	1	1	1	1	0	1
18a	M7288	Rectal	117	71	1	0	1	1	1	1	1	1	1	1	1	1	1	1	1	0	0	1	1	1	1	1	1	1	1	1	0	1
18b	M7559	Blood	117	71	1	1	1	1	1	1	1	1	1	1	1	1	1	1	1	1	1	1	1	1	1	1	1	1	1	1	1	1
19a	M6771	Rectal	80	1,065	1	1	1	1	1	1	1	1	1	1	1	1	1	1	1	0	0	1	1	1	1	1	1	1	1	1	1	1
19b	M6823	Blood	80	1,065	1	1	1	1	1	1	1	1	1	1	1	1	1	1	1	0	0	1	1	1	1	1	1	1	1	1	0	1
20a	M12098	Rectal	117	71	1	1	1	1	1	1	1	1	1	1	1	1	1	1	1	0	0	1	1	1	1	1	1	1	1	1	0	1
20b	M12270	Blood	117	71	1	0	1	1	1	1	1	1	1	1	1	1	1	1	1	0	0	1	1	1	1	1	1	1	1	1	1	1
21a	M8817	Rectal	80	1,065	1	1	1	1	1	1	1	1	1	1	1	1	1	1	1	0	0	1	1	1	1	1	1	1	1	1	0	1
21b	M10805	Blood	80	1,065	1	1	1	1	1	1	1	1	1	1	1	1	1	1	1	0	0	1	1	1	1	1	1	1	1	1	1	1
22a	M10864	Rectal	80	1,065	1	1	1	1	1	1	1	1	1	1	1	1	1	1	1	0	0	1	1	1	1	1	1	1	1	1	0	1
22b	M10900	Blood	80	1,065	1	1	1	1	1	1	1	1	1	1	1	1	1	1	1	0	0	1	1	1	1	1	1	1	1	1	0	1
23a	M6479	Rectal	117	71	1	1	1	1	1	1	1	1	1	1	1	0	1	1	1	0	0	1	1	1	1	1	1	1	1	1	0	1
23b	M9028	Blood	80	1,065	1	1	1	1	1	1	1	1	1	1	1	0	1	1	1	0	0	1	1	1	1	1	1	1	1	1	1	1
24a	M10520	Rectal	117	71	1	1	1	1	1	1	1	1	1	1	1	1	1	1	1	0	0	1	1	1	1	1	1	1	1	1	0	1
24b	M11630	Blood	117	71	1	1	1	1	1	1	1	1	1	1	1	1	1	1	1	0	0	1	1	1	1	1	1	1	1	1	1	1
25a	M12731	Rectal	117	71	1	0	1	1	1	1	1	1	1	1	1	1	1	1	1	0	0	1	1	1	1	1	1	1	1	1	1	1
25b	M13185	Blood	117	71	1	1	1	1	1	1	1	1	1	1	1	1	1	1	1	0	0	1	1	1	1	1	1	1	1	1	0	1
26a	M13032	Rectal	117	71	1	1	1	1	1	1	1	1	1	1	1	1	1	1	1	0	0	1	1	1	1	1	1	1	1	1	0	1
26b	M13661	Blood	80	1,065	1	1	1	1	1	1	1	1	1	1	1	1	1	1	1	0	0	1	1	1	1	1	1	1	1	1	1	1
27a	M12660	Rectal	80	1,065	1	1	1	1	1	1	1	1	1	1	1	1	1	1	1	0	0	1	1	1	1	1	1	1	1	1	0	1
27b	M13739	Blood	117	8,446	1	1	1	1	1	1	1	1	1	1	1	1	1	1	1	0	0	1	1	1	1	1	1	1	1	1	1	1
				Total	54	49	54	54	54	54	54	54	54	54	54	49	54	54	54	5	5	54	54	54	54	54	54	54	54	54	24	54

^*a*^ Entfm, *Enterococcus faecium*; Entls, *Enterococcus lactis*; hosp, hospital variant; com, community variant.

A comprehensive analysis of putative virulence markers (PVMs) revealed no disparities between colonization and infection isolates (Table 3). All isolates carried genes involved in adherence (e.g., *empA-empC*, *sgrA, ecbA, fms11, fms13, fms14),* biofilm formation, and colonization (*hylE* and *sagA*). Genes involved in carbohydrate metabolism, cell growth (e.g., *ccpA*, *orf1481*), and phosphotransferase systems (e.g., *ptsD*, *bepA*) were also found in all isolates. In contrast to the aforementioned results, *scm* was only present in approximately half of the colonization and half of the infection isolates (see also Table 3). *Scm* (second collagen adhesin) plays a role in adhesion and binds collagen type V and fibrinogen.

**Fig 2:**
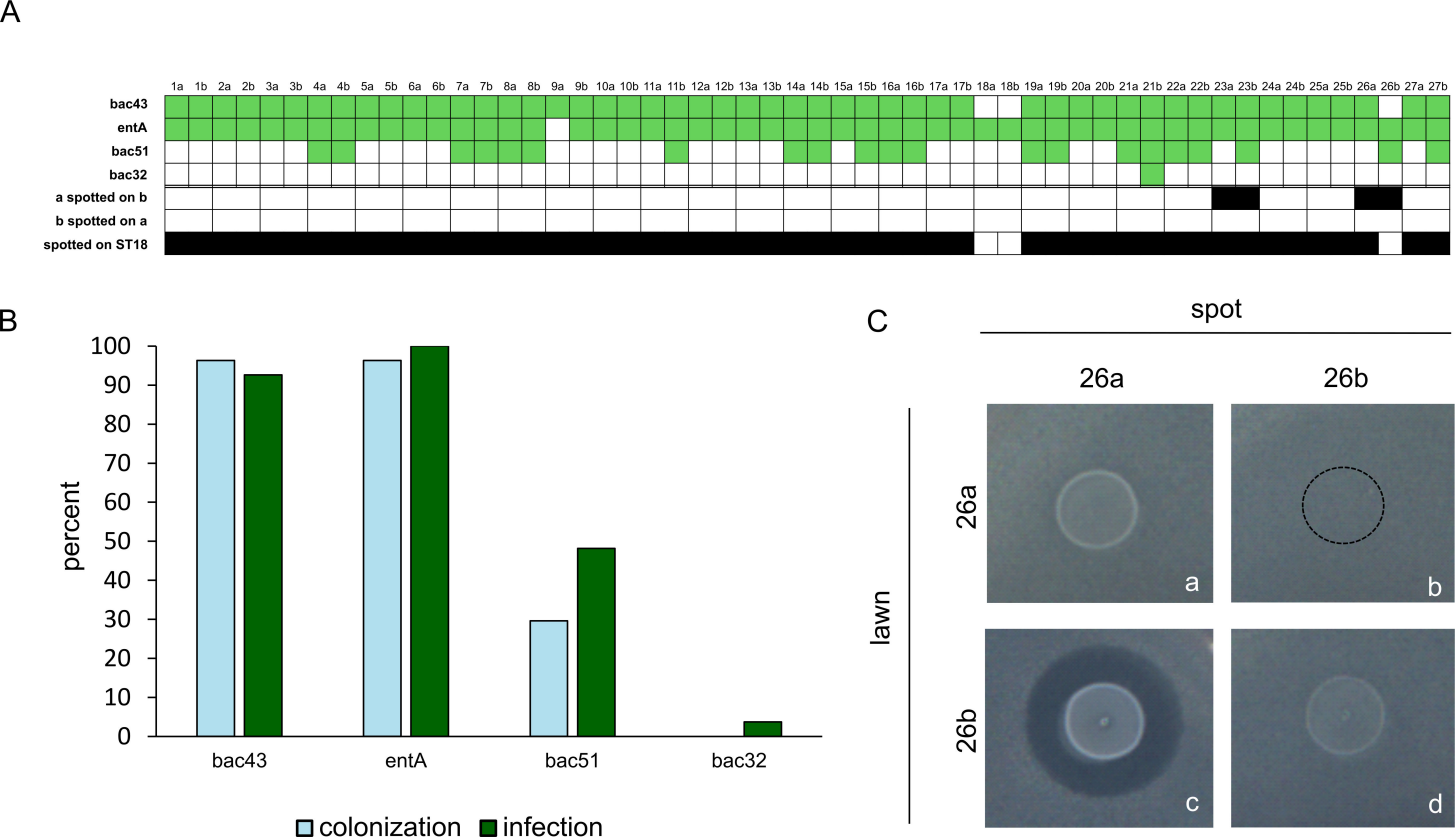
(**A**) Bacteriocins found in each isolate and results of the spot-on-lawn assay; bacteriocins detected are indicated in green. Colonization isolates are labeled a, infection isolates are labeled b. Observed growth inhibition in the spot-on-lawn assay is represented in black. (**B**) Percentage of all bacteriocin-encoding genes detected in colonization and infection isolates. (**C**) Representative image showing growth inhibition of a colonization isolate spotted on its corresponding infection isolate. A clear zone, indicating growth inhibition, was observed when the colonization isolate (26a) was spotted on its corresponding infection isolate (26b) (c). In contrast, spotting the infection isolate onto the colonization isolate resulted in minimal growth of the spot (b). Compared to spotting the colonization isolate onto itself, the spot is markedly reduced (a and d).

Bacteriocin screening identified four different bacteriocin-encoding genes in our isolates: *bac43*, *entA, bac51,* and *bac32*. Among these, *entA* was present in most isolates (98%), followed by *bac43* (94%), *bac51* (39%), and *bac32* (1%). In paired colonization and infection isolates, no differences in bacteriocin-encoding genes were found (Fig. 2B). Of all isolates encoding *bac51,* 85.7% belonged to ST80/CT1065.

### Interaction between colonization and infection isolates

In the spot-on-lawn assay, the infection isolates did not show any growth inhibition when they were spotted on their corresponding colonization isolate. Conversely, two colonization isolates inhibited the growth of their corresponding infection isolates (Fig. 2A). In these two cases (pair 23 and 26), the colonization isolate and infection isolate belonged to different STs (ST117/CT71 vs ST80/CT1065) (Fig. 2C). Furthermore, all isolates were spotted on an isolate belonging to ST18 as an internal control (7). Three isolates did not inhibit the growth of the historical ST18 (18a, 18b, 26b).

### Biofilm formation

**Fig 3:**
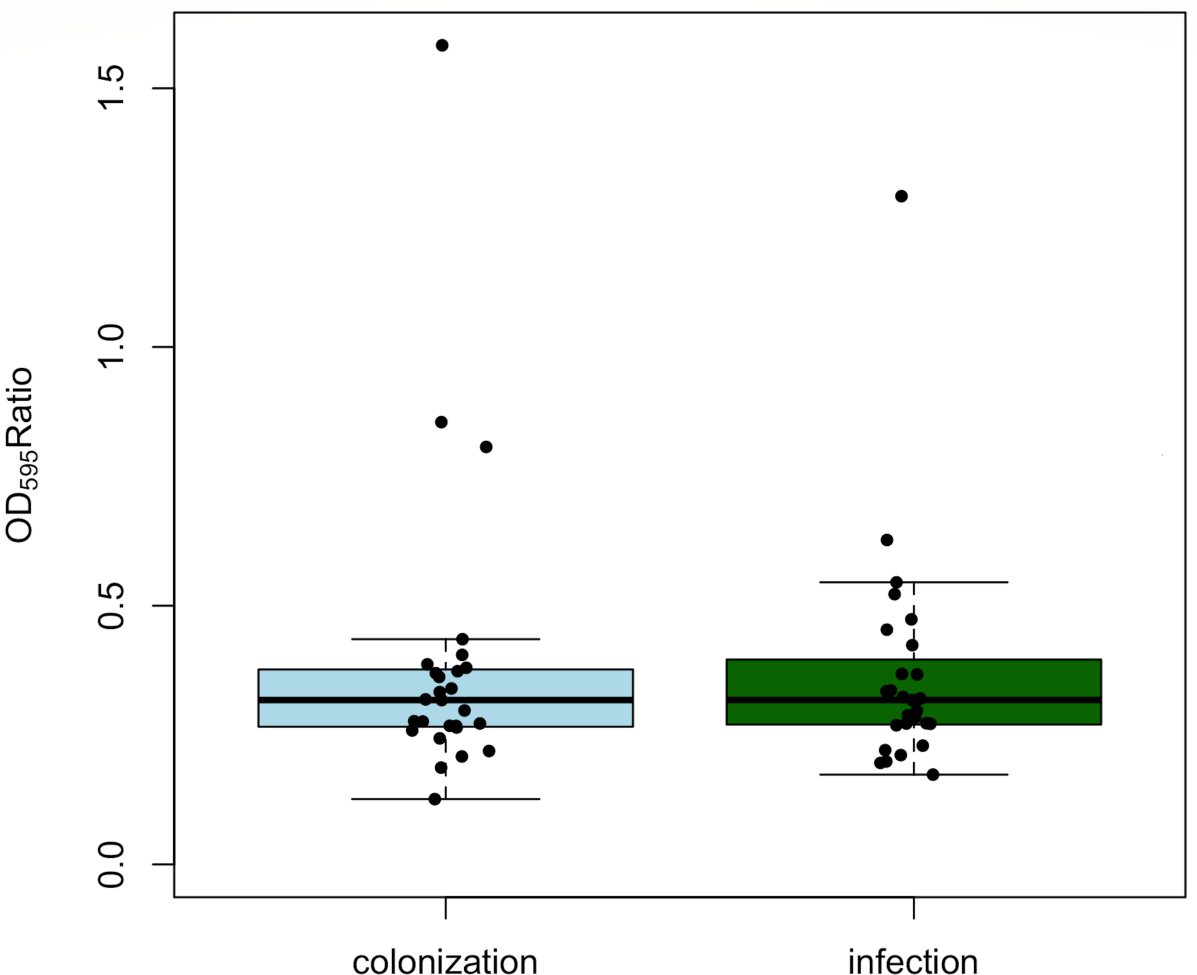
The boxplot diagram illustrates results of the biofilm formation assay showing the normalized OD_595_ ratio. No statistically significant difference was detected between the colonization (light blue) and infection (green) isolates of VREfm in terms of biofilm formation. Three colonization and two infection isolates were identified as outliers.

Biofilm formation of all isolates was measured with nine replicates on 3 different days (27 replicates in total). An overall limited ability to form biofilms in 96-well plates was observed. Colonization (normalized OD_595_ ratio 0.386) and infection (normalized OD_595_ ratio 0.370) isolates did not differ with respect to biofilm formation (*P* = 0.77) (Fig. 3). However, three colonization isolates (1a, 5a, 15a) and two infection isolates (12b, 25b) showed proportionally strong biofilm formation. These strains exhibited a deviation from the typical biofilm formation pattern observed in the remaining isolates, indicating potential variations in their capacity to produce biofilms.

## DISCUSSION

Individuals colonized with VREfm are at an increased risk of developing a subsequent VREfm infection. In this study, we aimed to elucidate the pathogenic factors of the isolates that initiate the transition from colonization to infection. We characterized 54 paired colonizing and invasive VREfm isolates and found no significant genetic and phenotypic differences between paired colonization and invasive isolates. This highlights the need for further studies that investigate the role of host factors in the development of BSI.

The present study included 27 VREfm pairs of patients initially colonized with VREfm who subsequently developed a BSI in a tertiary care setting in Germany. The majority of patients in the cohort were diagnosed with an oncological disease, particularly hematological-oncological disease, which has been previously described as a major risk factor for developing a VREfm infection (9). The gender and age distribution of the patients included was consistent with that of previous meta-analyses (10), showing a higher prevalence of VREfm BSI in males (11). Regarding ST distribution, ST117 and ST80 were identified as the predominant STs in our isolate collection, and all strains were *vanB* positive. This result is in line with data from the German National Reference Center for Staphylococci and Enterococci (NRC), which registered a shift from *vanA* to *vanB* in 2016 (12). Furthermore, data from the NRC and other studies confirm the predominance of the aforementioned STs not only in Germany but also worldwide (13–16). These STs have also been associated with a higher incidence of VREfm BSI, explaining the incidence of these STs in our cohort (11).

In recent years, several PVMs have been identified in *Enterococcus faecium* (17, 18). Previous studies have shown an enrichment of PVMs in hospital-adapted *E. faecium* strains, suggesting survival advantages in the hospital environment (6). We observed no differences in the number or type of PVMs between colonization and infection isolates and therefore conclude that VREfm does not acquire PVMs during colonization.

In addition to genomic analyses of the isolates, we investigated their ability to form biofilms. Our isolates produced only weak biofilms, which matched the results of previous studies (5, 19). Using RNAseq, Stege et al., however, observed, at a transcriptional level, an upregulation of genes related to biofilm and pili formation when VREfm were co-cultured with human-derived colonic epithelium (20). These findings highlight the importance of further investigation of pathogen-specific factors in the context of host-pathogen interactions.

In the spot-on-lawn assay, coexistence of colonization and infection strains was observed. Although infection isolates did not show direct inhibitory effects on the growth of colonization isolates, interspecific competition mediated by bacteriocins may nonetheless be an important factor contributing to the infection process. Ubeda et al. showed that an overgrowth of VREfm in the gastrointestinal tract leads to infection in an animal model (21). Thus, bacteriocins could help dominate other bacterial species in the gut, leading to subsequent infection. In our isolate collection, we identified enterocin A and bacteriocin 43 as the most prevalent bacteriocins. While most of our isolates inhibited the growth of historical ST18 VREfm, those isolates that did not carry *bac43* (18a, 18b, 26b) did not inhibit the growth of ST18 VREfm. These results confirm earlier findings that bacteriocin 43, also known as bacteriocin T8, is the main driver of the global dominance of ST117 and ST80 (7, 8). In the two cases (pair 23 and 26), when the colonization isolate (ST117) inhibited the growth of the corresponding infection isolate (ST80), the isolates belonged to different STs. In pair 26, the observed growth inhibition can be explained by the absence of *bac43* in the infection isolate (26b). In pair 23, *bac43* was detected in both the colonization and the infection isolate. This observation raises the question of whether another factor besides bacteriocin 43 could be important in growth inhibition.

Despite the strengths of this study, there are several limitations that need to be acknowledged. As only first colonization and first BSI VREfm isolates were investigated, the message of the current study might be limited. Furthermore, time points between first detection of VREfm colonization and the development of an infection were highly variable. Despite this, in the interval between rectal detection and the onset of infection, paired isolates displayed no phenotypic or genotypic variations, indicating that the within-host evolution is not a critical factor for the development of VREfm BSI.

In summary, our study revealed only minor differences at the genomic and phenotypic level between colonizing and infecting isolates. This suggests that the initial clone that colonizes a patient subsequently causes infection without acquiring virulence during colonization. Therefore, other factors, such as the intestinal barrier and the interaction of host and pathogen, should be investigated to shed light on the transition from colonization to BSI of VREfm. The fact that oncology and hematology-oncology patients are often treated with chemotherapy, resulting in a barrier breach, highlights the importance of investigating how VREfm crosses the gastrointestinal barrier. We also recommend that infection prevention measures should be implemented to prevent VREfm colonization in at-risk patients, while minimizing the risk of developing an infection. In addition, in the absence of decolonization strategies, risk assessment should be introduced to help identify patients at risk of developing VREfm BSI.

## MATERIALS AND METHODS

### Bacterial strain collection

In this study, 54 paired VREfm isolates were included, obtained between 2015 and 2023 at a tertiary hospital in Germany. One pair consisted of a rectal isolate and a subsequent BSI isolate from the same patient.

### Study design

The 1,438-bed University Hospital Würzburg is a tertiary care center admitting approximately 60,000 patients per year. According to national guidelines (22), weekly and admission VREfm screening of patients is performed on defined high-risk wards, namely on hematology-oncology, intermediate care, and intensive care units. In case of VRE detection during screening, basic hygiene measures are proceeded with, while contact precautions or isolation strategies are not implemented in single cases. In case of epidemiological connected cluster settings, outbreak management and bundle strategies are implemented, including intensified surface disinfection, contact precautions, and patient isolation.

### Microbiological culturing, antibiotic susceptibility testing

Rectal swabs or positive blood cultures were plated on blood agar plates and incubated for 24 h at 36°C. Species were differentiated by VitekMS (bioMérieux, Marcy l'Etoile, France). Susceptibility testing with Vitek2 (bioMérieux) or by using agar gradient diffusion (Liofilchem, Roseto degli Abruzzi, Italy) was interpreted according to breakpoints of the European Committee on Antimicrobial Susceptibility Testing.

### Whole-genome sequencing-based typing and virulence factor analysis

Isolated VREfm were subjected to whole genome sequencing using the Illumina NextSeq 2000 platform (Illumina Inc., San Diego, CA, USA). After d*e novo* assembly using Velvet (23), SeqSphere^+^ software version 10.0.5 (Ridom, Münster, Germany) was used for a comparison of coding regions in a gene-by-gene approach using the previously published cgMLST target scheme for *E. faecium* (24). Single nucleotide variant analysis of sequenced strains was performed using the same software. To display the genetic relationship of genotypes, the minimum spanning tree algorithm was applied (Fig. 1; Fig. S1), while isolates differing in three cgMLST target genes or less were defined as closely related (Fig. 1). MLST STs as well as cgMLST complex types were extracted from whole-genome sequencing data *in silico*. PVMs were identified using the VirulenceFinder of the Center for Genomic Epidemiology with a selected threshold of >98% (25, 26). To screen for bacteriocin-encoding genes, a recently published database with a selected threshold of >80% and >60% coverage was used in CGE MyDbFinder (27).

### Biofilm formation assay

The capacity of the clinical isolates to form biofilms on abiotic surfaces was evaluated using the semi-quantitative assay previously described (19). In brief, an overnight culture of VREfm in tryptic soy broth (TSB; Becton Dickinson, Heidelberg, Germany) was prepared, the OD_600_ was adjusted to 0.35, and subsequently diluted 1:10 with TSB. These suspensions were loaded into a 96-well plate (cell culture plate, 96-well, polystyrene, Sarstedt, Nümbrecht, Germany) at a final volume of 200 µL and incubated for 48 hours at 36°C under static conditions. The supernatant was discarded, and the plates were washed three times with 200 µL PBS. Plates were heat-fixed at 56°C for 1 hour and stained for 5 minutes with 1% crystal violet. After staining, plates were rinsed with deionized water, dried, and crystal violet was dissolved in 200  µL of a 20:80 acetone/ethanol mixture. OD_595_ was measured spectroscopically (Thermo Scientific Multiskan EX, Thermo Fisher Scientific, Waltham, MA, USA). *E. faecalis* ATCC 29212 was used as a positive control.

### Spot-on-lawn assay

To better understand the interaction between colonization and infection isolates, a spot-on-lawn assay was performed. Overnight cultures of colonization isolates in brain heart infusion (BHI; Becton Dickinson, Heidelberg, Germany) were adjusted to OD_600_ = 0.05. One hundred microliters of the previously prepared suspension was added to 5 mL of liquefied 0.7% BHI agar, mixed, and poured on 1.5% BHI agar. Five microliters of the overnight culture of the corresponding infection isolates was spotted onto the lawn and vice versa. The plates were then incubated for 18–24 hours at 36°C. The sample was considered positive if the zone of inhibition was >1 mm. A historical VREfm (MLST ST18) was used as a control strain (7).

### Computational and statistical analysis

For biofilm formation assays, on each measurement day, three 96-well plates were identically prepared with three technical replicates, including negative (blank) and positive control. The OD_595_ measurements of biofilm formation were averaged per sample, and the average negative control (blank) value was subtracted. The resulting values were normalized by dividing by the average positive control value to control for daily variations in biofilm formation. Finally, the normalized OD_595_ ratios of each sample were averaged across the well plates. This was done for each measurement day, and the resulting three OD_595_ measurements were again averaged across measurement days. A paired two-tailed *t*-test was performed to test for statistical significance using R Studio (R version 4.4.2) (The R Foundation, Vienna, Austria). For association analysis of virulence factors with clinical outcome, Fisher’s exact test was performed. Statistical significance was assumed at *P* < 0.05.

## Data Availability

Whole genome sequencing data of analyzed VRE genomes were submitted to the NCBI database (PRJNA1262308).

## SUPPLEMENTAL MATERIAL

Figure S1 spectrum.01750-25-s0001.pdf Minimum spanning tree of VREfm colonization and infection isolates.

Table S1 spectrum.01750-25-s0002.docx Genomic analysis metrics.

ASM does not own the copyrights to Supplemental Material that may be linked to, or accessed through, an article. The authors have granted ASM a non-exclusive, world-wide license to publish the Supplemental Material files. Please contact the corresponding author directly for reuse.
