# Supplementary material for: Comparative analysis of vancomycin-resistant enterococci in colonization and infection—a longitudinal study
Source: Microbiol Spectr. 2025 Oct 16;13(11):e01750-25. doi: 10.1128/spectrum.01750-25 (PMC12584739; doi:10.1128/spectrum.01750-25)
Supplement: Figure S1 — Minimum spanning tree of VREfm colonization and infection isolates. [file spectrum.01750-25-s0001.pdf]

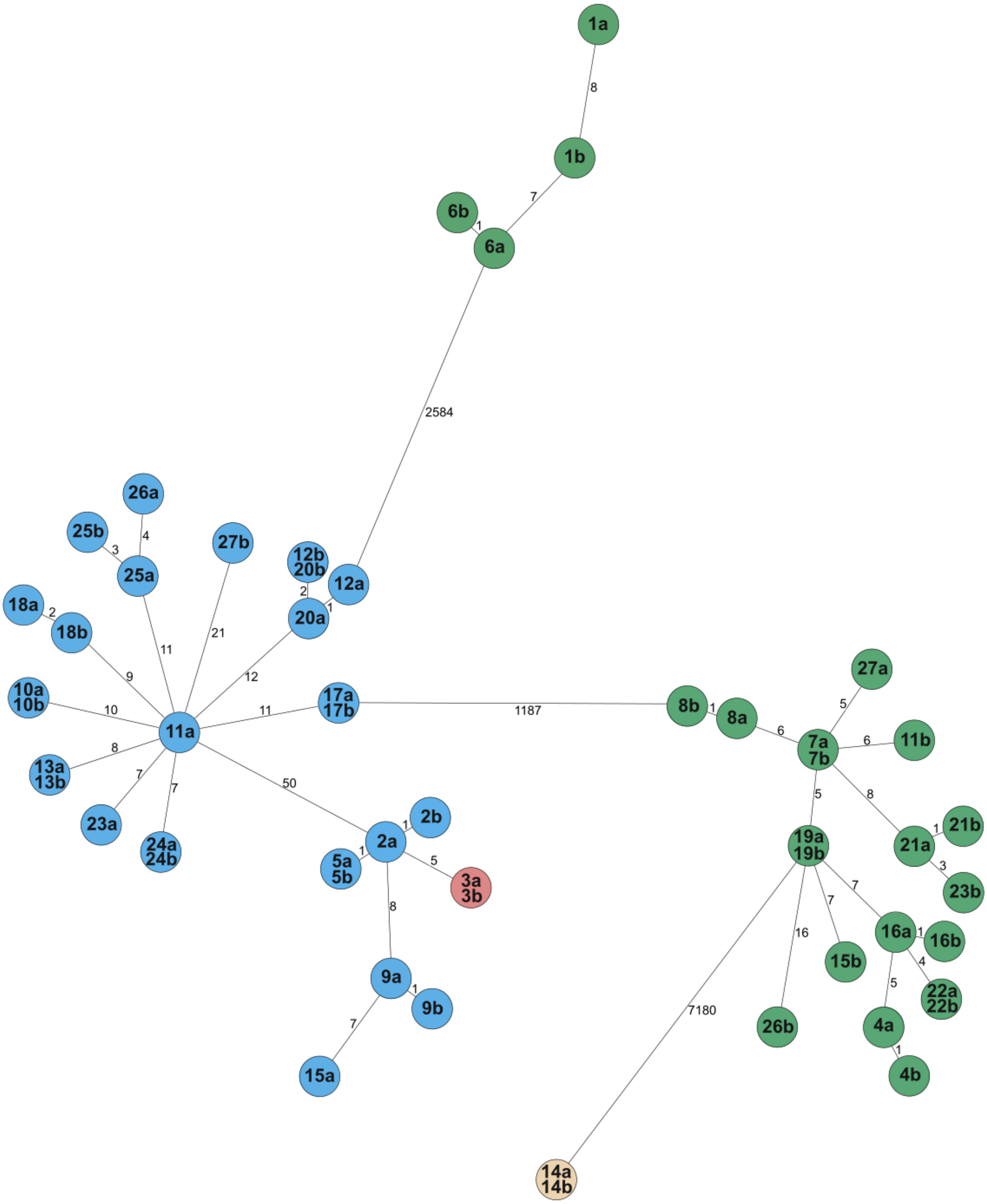

Supplementary Figure 1: Minimum Spanning tree of VREfm colonization (labelled a) and infection (labelled b) isolates displaying results of the single nucleotide variant (SNV) analysis, based on 16,171 nucleotide columns, pairwise ignoring missing values. Circle colours represent *in silico* extracted Multilocus Sequence Types Sequence Types (STs) and are identical to Figure 1 of the main manuscript. The number of nucleotides differing is given on connecting lines.
