# Supplementary material for: Comparative analysis of vancomycin-resistant enterococci in colonization and infection—a longitudinal study
Source: Microbiol Spectr. 2025 Oct 16;13(11):e01750-25. doi: 10.1128/spectrum.01750-25 (PMC12584739; doi:10.1128/spectrum.01750-25)
Supplement: Table S1 — Genomic analysis metrics. [file spectrum.01750-25-s0002.docx]

Supplementary Table I: Genomic analysis metrics

| **Patient** | **Strain** | **Contig Count (Assembled)** | **N50 (Assembled)** | **Read Count (Assembled)** | **Assembly Base Count** | **Max Contig Length (Assembled)** | **Min Contig Length (Assembled)** | **Avg. Contig Length (Assembled)** | **Avg. Coverage (Assembled)** | **Read Base Count (Assembled)** | **cgMLST % Good Targets** |
| --- | --- | --- | --- | --- | --- | --- | --- | --- | --- | --- | --- |
| 1a | M5324 | 280 | 29084 | 1929078 | 2996572 | 130228 | 141 | 10702 | 95 | 284444974 | 98.5 |
| 1b | M7369 | 326 | 25659 | 1567128 | 3036544 | 106723 | 137 | 9314 | 76 | 232223911 | 97.6 |
| 2a | M4923 | 265 | 37524 | 2136290 | 3026031 | 112907 | 137 | 11418 | 105 | 317634014 | 99.2 |
| 2b | M12038 | 307 | 24397 | 1836784 | 3019534 | 113020 | 137 | 9835 | 90 | 272696235 | 98.7 |
| 3a | M11253 | 308 | 27527 | 2005508 | 3020928 | 107408 | 137 | 9808 | 98 | 296578089 | 98.4 |
| 3b | M11278 | 297 | 29234 | 1857072 | 3024873 | 107408 | 137 | 10184 | 91 | 274829701 | 98.4 |
| 4a | M10885 | 306 | 33401 | 2068716 | 3038152 | 107369 | 137 | 9928 | 102 | 308880578 | 98.6 |
| 4b | M11870 | 317 | 32675 | 2408650 | 3038561 | 130228 | 141 | 9585 | 117 | 355999149 | 98.6 |
| 5a | M9308 | 312 | 24923 | 2044678 | 2994981 | 106724 | 137 | 9599 | 101 | 302684820 | 98.2 |
| 5b | M9368 | 305 | 25428 | 1672536 | 2999943 | 81805 | 137 | 9835 | 83 | 247653627 | 98.5 |
| 6a | M5550 | 325 | 29245 | 1852858 | 3101372 | 107409 | 137 | 9542 | 88 | 273268962 | 98.2 |
| 6b | M5974 | 301 | 29553 | 2184670 | 3086567 | 107409 | 137 | 10254 | 105 | 324996679 | 98.5 |
| 7a | M7056 | 312 | 29233 | 1919788 | 3038605 | 104375 | 137 | 9739 | 94 | 285562391 | 98.6 |
| 7b | M7132 | 269 | 34909 | 2477092 | 3039009 | 130224 | 137 | 11297 | 121 | 367077371 | 98.9 |
| 8a | M12355 | 314 | 26904 | 1888140 | 3010838 | 88784 | 137 | 9588 | 93 | 279345627 | 98.3 |
| 8b | M12375 | 294 | 30058 | 2047520 | 3006310 | 106725 | 141 | 10225 | 100 | 302002535 | 98.3 |
| 9a | M3740 | 426 | 26060 | 1795032 | 3086027 | 100842 | 137 | 7244 | 86 | 266657324 | 98.5 |
| 9b | M10251 | 388 | 26846 | 1646272 | 3046052 | 84345 | 137 | 7850 | 80 | 245073830 | 98.6 |
| 10a | M11988 | 308 | 32645 | 2044816 | 3043561 | 108551 | 137 | 9881 | 100 | 303200560 | 98.5 |
| 10b | M12072 | 311 | 29252 | 2276800 | 3043881 | 120374 | 137 | 9787 | 112 | 339409543 | 98.9 |
| 11a | M6985 | 327 | 33354 | 1891618 | 3060133 | 85142 | 137 | 9358 | 92 | 280824010 | 98.3 |
| 11b | M8421 | 346 | 30820 | 2514366 | 3043432 | 106721 | 137 | 8796 | 123 | 374324835 | 98.7 |
| 12a | M12188 | 298 | 31037 | 2010002 | 3028220 | 98564 | 137 | 10161 | 99 | 298489370 | 98.7 |
| 12b | M12247 | 300 | 30762 | 3394152 | 3018126 | 104030 | 137 | 10060 | 167 | 504864761 | 98.9 |
| 13a | M9079 | 283 | 33674 | 2019366 | 3019948 | 112907 | 137 | 10671 | 99 | 299949116 | 98.8 |
| 13b | M9112 | 299 | 32671 | 1820034 | 3019322 | 100841 | 137 | 10098 | 90 | 270959798 | 98.8 |
| 14a | M8362 | 303 | 34528 | 1840902 | 3036884 | 217214 | 137 | 10022 | 89 | 271635103 | 98.2 |
| 14b | M8392 | 241 | 40518 | 2229034 | 3028298 | 353920 | 141 | 12565 | 109 | 330474990 | 98.8 |
| 15a | M3255 | 306 | 29234 | 1918776 | 3014710 | 112906 | 137 | 9851 | 94 | 284654799 | 98.7 |
| 15b | M7568 | 293 | 32370 | 2241168 | 3008758 | 107408 | 137 | 10268 | 110 | 331941465 | 98.7 |
| 16a | M11222 | 402 | 19420 | 1475214 | 3029271 | 60550 | 137 | 7535 | 72 | 218842715 | 95.6 |
| 16b | M11514 | 328 | 34709 | 2292042 | 3042358 | 112911 | 141 | 9275 | 112 | 339516891 | 98.7 |
| 17a | M8820 | 290 | 31365 | 1948450 | 3034600 | 85016 | 141 | 10464 | 94 | 286565066 | 98.7 |
| 17b | M8856 | 294 | 32671 | 1593672 | 3046945 | 85618 | 137 | 10363 | 78 | 236219963 | 98.6 |
| 18a | M7288 | 388 | 19466 | 1436654 | 2778498 | 60427 | 125 | 7161 | 64 | 179012867 | 97.3 |
| 18b | M7559 | 277 | 35035 | 2053074 | 3060804 | 107408 | 137 | 11049 | 100 | 305068461 | 98.9 |
| 19a | M6771 | 334 | 29234 | 1881222 | 3036659 | 113014 | 137 | 9091 | 92 | 279612112 | 98.4 |
| 19b | M6823 | 290 | 33862 | 2039580 | 3035928 | 112907 | 137 | 10468 | 100 | 303871534 | 98.8 |
| 20a | M12098 | 302 | 30882 | 1898140 | 3025694 | 85039 | 137 | 10018 | 93 | 281309393 | 98.5 |
| 20b | M12270 | 286 | 33284 | 2941632 | 3025531 | 107412 | 141 | 10578 | 144 | 434256112 | 98.7 |
| 21a | M8817 | 284 | 32671 | 1727432 | 3038660 | 106721 | 137 | 10699 | 85 | 257194063 | 98.5 |
| 21b | M10805 | 383 | 30116 | 1666210 | 3125341 | 106725 | 141 | 8160 | 79 | 246439935 | 98.7 |
| 22a | M10864 | 324 | 34483 | 1777822 | 3041970 | 130224 | 137 | 9388 | 87 | 264496255 | 98.4 |
| 22b | M10900 | 288 | 35695 | 1868686 | 3042061 | 107405 | 137 | 10562 | 91 | 277431320 | 98.8 |
| 23a | M6479 | 302 | 32671 | 2082990 | 3028434 | 106236 | 137 | 10027 | 103 | 310640093 | 98.9 |
| 23b | M9028 | 284 | 34735 | 2094980 | 3122398 | 107464 | 137 | 10994 | 99 | 310294945 | 98.9 |
| 24a | M10520 | 278 | 33401 | 2171008 | 3060549 | 107408 | 137 | 11009 | 105 | 322639648 | 98.9 |
| 24b | M11630 | 345 | 32355 | 1962474 | 3060430 | 101484 | 137 | 8870 | 96 | 292557151 | 98.5 |
| 25a | M12731 | 303 | 29621 | 2477070 | 3016992 | 100834 | 129 | 9957 | 111 | 334677202 | 98.7 |
| 25b | M13185 | 309 | 32366 | 2275844 | 3061625 | 100833 | 129 | 9908 | 101 | 308224430 | 99.0 |
| 26a | M13032 | 296 | 34803 | 2011340 | 3063270 | 94715 | 141 | 10348 | 97 | 298418611 | 99.0 |
| 26b | M13661 | 306 | 33401 | 1879206 | 3034389 | 130223 | 137 | 9916 | 92 | 278170120 | 98.7 |
| 27a | M12660 | 265 | 33972 | 2338328 | 3040363 | 106725 | 141 | 11473 | 114 | 345683517 | 98.9 |
| 27b | M13739 | 310 | 34909 | 1535524 | 3044194 | 110583 | 137 | 9819 | 75 | 227210189 | 98.3 |
